# Life chances after surgery of congenital heart disease: A case-control-study of inter- and intragenerational social mobility over 15 years

**DOI:** 10.1371/journal.pone.0246169

**Published:** 2021-02-19

**Authors:** Siegfried Geyer, Katharina Fleig, Kambiz Norozi, Lena Röbbel, Thomas Paul, Matthias Müller, Claudia Dellas

**Affiliations:** 1 Medical Sociology Unit, Hannover Medical School, Hannover, Germany; 2 Department of Paediatrics, Western University, London, Ontario, Canada; 3 Dept. of Paediatric Cardiology, Intensive Care and Pneumology, University Medical Center, Göttingen, Germany; 4 Department of Paediatric Cardiology and Intensive Care Medicine, Hannover Medical School, Hannover, Germany; 5 Department of Hematology, Hemostasis, Oncology and Stem Cell Transplantation, Hannover Medical School, Hannover, Germany; University of Luxembourg, LUXEMBOURG

## Abstract

**Background:**

Patients of congenital heart disease surgery have good prospects for reaching old age. Against the backdrop of increasing life expectancies, the question of how well such patients are mastering daily routines and their working life emerges. In our study, the educational and occupational performance of patients over 15 years was examined.

**Methods:**

Intergenerational social mobility (changes in social positions from the parental generation to the generation of children) was examined in terms of education, and intragenerational social mobility (changes in positions within the same generation, i.e., in individuals over their life courses) was examined in terms of occupational positions. Comparisons were made between patients and a control group drawn from the German Socio-Economic Panel (SOEP). Controls were drawn from respondents who participated in the 2004 and 2018 SOEP surveys.

**Results:**

The data were from 244 out of 360 patients (68%) with complete social data from the first survey (2003–2004) and who were included in the follow-up (2017–2019), and 238 controls were drawn from the SOEP. At the time of the second survey, subjects’ ages ranged from 28 to 59 years of age (M = 40.1 years). Intergenerational educational mobility did not differ between cases and controls. For intragenerational social mobility, downward changes were more frequent among controls. This latter finding may be explained by patients retiring earlier than the general population. Retirement rates increased over time, particularly among patients with severe congenital malformations. Unemployment rates were also higher among patients.

**Conclusions:**

Taken together, although a considerable proportion of patients with congenital heart disease retired prematurely or never entered the labour force, their educational and occupational careers proceeded more favourably than expected.

## Introduction

The primary challenge for patients with congenital heart disease has long been to survive, but today, approximately 90% of such patients reach adulthood [1], and this also applies to patients with complex malformations [2]. Thus, it is important to understand how they survive and what life opportunities surgery opens up for them from a long-term perspective [3, 4]. A recent study reported that patients with all types of congenital heart diseases (CHDs) suffered from considerable cardiovascular and non-cardiovascular morbidity and from medication-related morbidity [2, 4, 5]. It can be concluded that the everyday life of patients is impaired by the concomitants of the disease [6–8]. Against this backdrop of persisting morbidity, it needs to be known how well patients are passing through their school education what in turn is an important prerequisite for integration into the labour market. Findings on this topic are contradictory and dependent on study design as well as on the subpopulations considered. A US-based study with N = 334 children with CHD reported that more individuals with CHD than without were in need of special assistance [9]. In a study of siblings, children with CHD had lower school performance and more cognitive impairments than their siblings without malformations [10]. A study from the Netherlands compared the educational levels of 1,496 CHD patients under 40 years of age with a group of N = 6,810 controls. Patients were more likely to reach lower educational levels than the controls, but the differences between groups were small for less severe forms of CHD and more pronounced for complex types of malformations [11]. In contrast, a register-based survey study with n = 1,198 women and men with all types of CHD reported that 59.4% of patients had reached a high educational level [12]. In a Finnish study, the educational levels of patients were comparable or higher than expected relative to the general population. Comparisons were not performed with matched controls but with national register-based data [13]. A recent French study with n = 135 CHD patients concluded that they had achieved lower educational levels than the average member of the French population, but again, the authors did not directly compare patients’ achievements with those of controls [14].

School education has a placement function for occupational positions, as most occupations require certain educational levels; i.e., educational prerequisites are usually increasing with occupational position. After having completed schooling, individuals have to decide whether to enter the labour market, and then they must consider how long they are able to remain economically active despite facing increasing risks of premature health impairments. A cross-sectional survey study covering n = 4028 adults with CHD from 15 countries reported employment rates between 43 and 80%, depending on the country of origin. The lowest employment and the highest unemployment rates were reported for India and Argentina, and the most favourable figures were reported for Belgium [15]. If the employment rates of CHD patients are considered within countries, the findings are also heterogeneous. A Finnish study published in 2003 used nationwide data from 2896 CHD patients, and it was reported that the employment rate in this group was higher than in the general population [13]. A Danish study examined the consequences of Tetralogy of Fallot, a complex malformation, for employment status. The authors emphasized that patients earned high-level educational degrees, but their average employment level was lower and retirement rates were higher than expected [16]. A Japanese study also reported that patients were at a higher risk of unemployment than the general population, but only a minority of jobless individuals attributed it to their disease [17].

In summary, evidence on the educational and occupational performance of CHD patients is inconsistent. While studies on education point towards more favourable outcomes, the results of analyses on employment are heterogeneous, permitting the conclusion that patients may attain lower positions than their counterparts from the average population. International comparisons are impeded by country-specific policies towards the integration of people with impairments and by different proportions of CHD-types. Finally, only a few studies used control groups or longitudinal designs.

In our own study, a different approach was applied; social background was used as a point of departure for studying social mobility in comparison with the general population from a longitudinal perspective. The first wave of the study was conducted from 2003–2004, and it was found that up- and downward changes in the occupational positions of patients compared with the positions of their parents did not differ from those of the general population [18]. However, more patients than controls were employed part-time, and a small fraction never entered the labour market [19]. The present paper builds on these earlier findings by extending the observation period through the use of education for studying intergenerational mobility and the use of intragenerational occupational mobility endpoints.

The following research questions are addressed:

1. Does social mobility in terms of upward or downward changes in social positions from parents to children (i.e., intergenerational social mobility) differ between patients and controls from the general population in terms of educational level?
2. Do the rates of retirement and unemployment differ between patients and controls?
3. Do changes in occupational positions for the same individuals over time (intragenerational occupational mobility) differ between patients and controls?

**Fig 1:**
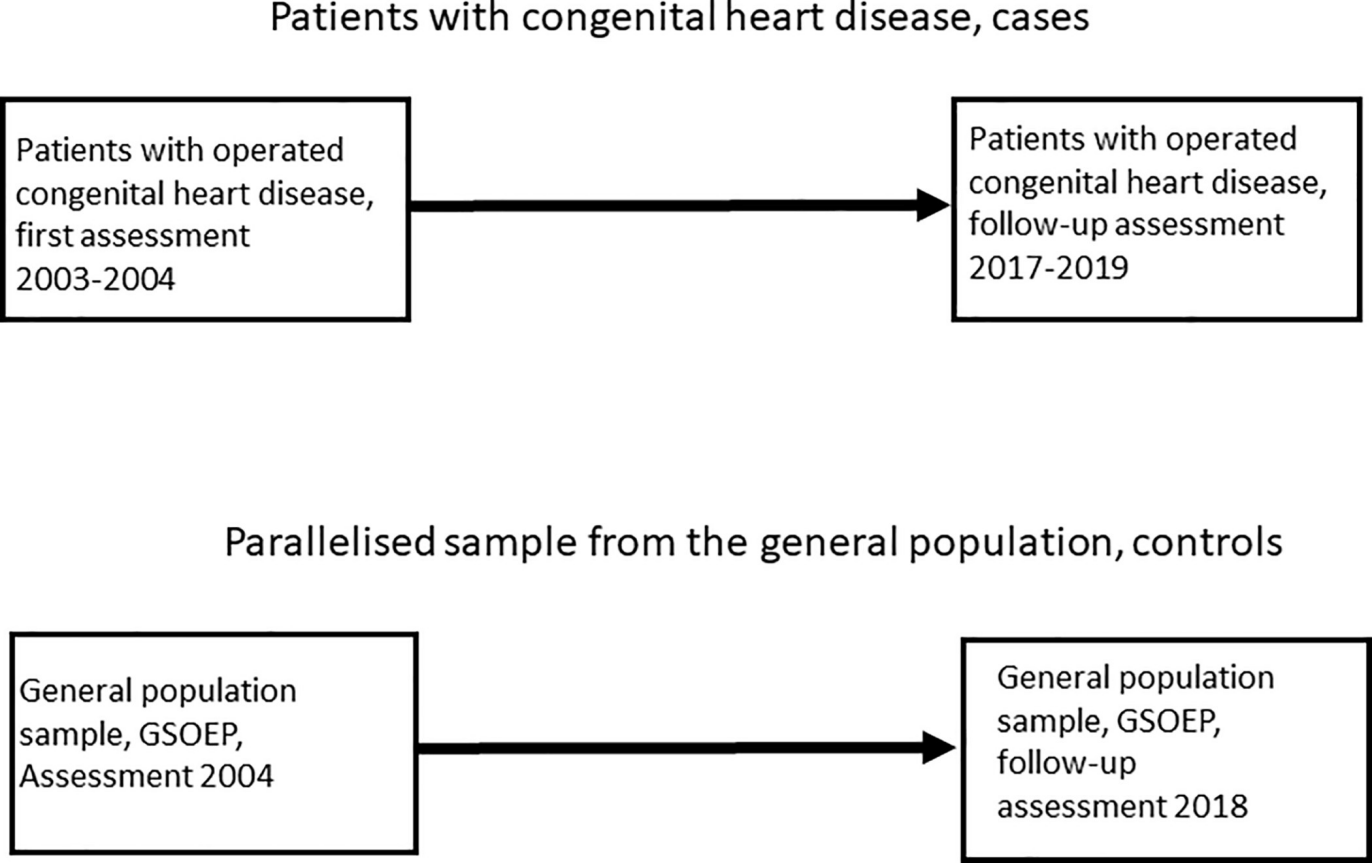
Study design for examining inter- and intragenerational social mobility. Cases (n = 244) and controls (n = 238) were matched according to parental education, subject gender and subject age.

We used data from a longitudinal study of patients with congenital heart disease. The first survey was conducted from 2003 to 2004, and the second was conducted from 2017 to 2019. These data were compared with those from two waves of the German Socio-Economic Panel (SOEP) conducted in 2004 and in 2018. The resulting design is summarized in Fig 1.

This study is not confined to a single type of malformation but includes the whole range of CHD types, covers a long follow-up period, combines medical and social data, and uses a case-control design that permits the separation of disease-specific developments from those occurring in the whole population.

## Materials and methods

Survey questions and categories for depicting socioeconomic positions and occupations were adopted from the German Socio-Economic Panel (SOEP) [20, 21] to permit direct comparisons of patients and controls. The questionnaires were published in English and German and can be downloaded from https://www.diw.de/de/diw_01.c.32045.de/frageb_ouml_gen.html.

**Patients** from the University Medical Centre Göttingen (Germany) were invited to participate in this study and to give informed consent in writing. This hospital is unique because CHD patients are continuously cared for even after reaching adolescence; thus, complete health records were available. This made it possible to estimate the systematic effects of non-response and of deaths. Findings on comparisons between responders and non-responders in the first wave were published in an earlier paper [22]. Patients participating in the first survey were examined from May 2003 to June 2004, and the second survey was conducted from May 2017 to April 2019. As the study was designed to examine social mobility and occupational careers, patients with syndromes associated with impaired cognitive ability were excluded [22]. Patients were interviewed in the hospital by means of standardized personal interviews to assess the social situation of the patients and their parents. The cardiologic examinations consisted of taking medical histories, performing medical examinations, taking laboratory measures and conducting spiroergometric examinations to obtain information on cardiac status. Based on the type of surgery and the severity of the congenital malformation, patients were classified into three types [23]: those who could be *curatively* operated on with or without minor residual defects, those with *reparative* surgery to establish normal heart function with residual symptoms, and those with *palliative* surgery, indicating permanent impairment and continuously perceptible symptoms.

**Controls** were drawn from the German Socio-Economic Panel (SOEP) [20, 21]. The SOEP is a national survey for monitoring social development and social change that started in 1984 with annual follow-ups. To establish and maintain representativity with respect to defined population characteristics, attrition is compensated for by drawing new subjects in refresher samples. Analogous to the years when patients were examined, controls were drawn from respondents who had participated both in 2004 and in 2018 to compare the long-term courses of their educational and occupational careers. The selection of controls took place by using parental education and the age and gender of the respondents as matching criteria. This procedure should ensure that the initial social standings of cases and controls were identical and that the findings for cases and controls are comparable. The matching procedure was performed in four steps:

Step 1: The data from the 2004 and 2018 SOEP waves were merged into a longitudinal dataset by using the unique person number as identifier.

Step 2: All SOEP cases less than 28 years of age or older than 59 years of age (during the survey 2018) were excluded.

Step 3: Only SOEP cases who participated in surveys 2004 **and** 2018 were kept.

Step 4: Multivariate matching of patients and controls was performed by using patients as points of reference. Fathers’ educational level (if unavailable, the educational level of mothers), gender of patients/controls, and age of patients/controls were used as matching variables.

### Ethics approval

The first part of this study was reviewed and approved by the ethics committee of Hannover Medical School under no. 3710 (date: 04-10-2004) and by the Göttingen University Clinic under no. 10/2/01 (date: 01-03-2001). The second part was reviewed and approved by the ethics committee of the University of Göttingen under no. 15/8/14.

### Variables used in the statistical analyses

**Educational level** was collected for patients/survey respondents and for parents. It was classified into “no formal educational qualification achieved”, “8/9 years of school education”, “10 years of school education”, and “12/13 years of school education”. In Germany, the maximum number of years of school education is 12 or 13 years, depending on the federal state. For parents, educational level was assigned according to the father’s school education; if unavailable, the mother’s education was assigned. Differences between father’s and mother’s education are not assumed to bias our results because inspecting the available information led to a high concordance of qualifications, which in turn supports the practice described.

**Occupational level** was based on the International Standard Classification of Occupations, versions 1988 (ISCO88) [24] and 2008 (ISCO08) [25]. This is an internationally used hierarchically ordered classification of 436 occupations, and translations of the older into the most recent classification system were performed by using correspondence tables issued by the International Labour Office [25]. By aggregating occupational groups, a four-category classification of skill levels was created:

**Skill level 1**: Manual and repetitive activities requiring physical effort and perseverance including simple calculational, reading and writing capabilities. Examples of occupations in this category are office cleaners, freight handlers, garden labourers or kitchen assistants [25] (p.12).

**Skill level 2**: Activities requiring manual skills, flexibility in dealing with people or handling goods and operating machines. Some mathematical and technical skills and flexibility are necessary for reacting flexibly or for coping with new situations in writing and/or verbally. Examples are bus drivers, secretaries, account clerks, sewing machinists, dressmakers, shop sales assistants, police officers or motor vehicle assistants [25] (p.12).

**Skill level 3**: Occupations requiring specialized knowledge for coping with complex situations and tasks. Communication and coordination skills, advanced mathematical skills and the ability to communicate complex topics are required. Examples are shop managers, medical laboratory technicians, commercial sales representatives, diagnostic medical radiographers or computer support technicians [25] (p.13).

**Skill level 4**: Occupations requiring complex decision making and planning, advanced problem-solving strategies, mathematical and oral capabilities and the ability to acquire new and complex contents. Examples are sales and marketing managers, civil engineers, secondary school teachers, medical practitioners, operating theatre nurses or computer systems analysts [25] (p.13).

**Social mobility** was examined by sorting educational and occupational levels ordinally and by calculating differences between starting and ending positions. For intergenerational mobility in terms of education, this refers to changes in levels from the parental to the subsequent generation. Intragenerational mobility refers to changes in occupational levels for the same individual over time by comparing starting and ending positions. The resulting order has a midpoint scaled as “0”, denoting the absence of social mobility and a maximum of three upward (+1/+2/+3) or downward changes (-1/-2/-3). Only a few cases were at the extreme ends of the mobility distributions (see Table 1). To avoid estimation problems arising due to small numbers, categories were collapsed in order to restrict the regression analyses to upwardly or downwardly mobile or to stable individuals.

**Table 1:** Basic distributions of the variables used in the analyses.

	Gender Frequencies/col- %			Mean age/ Sd in 2018		Type of surgery (patients only)						
Frequency %	Women	Men		Women	Men	Curative	Reparative			Palliative	Missing	Total
Patients	102 41.8%	142 58.2%		39.6 yr./ 8.6	40.0 yr./ 8.6	44 18.0%	164 67.2%			36 14.8%	0	244 100%
Controls	97 40.8%	141 59.2%		40.4 yr./ 8.0	40.5 yr./ 7.8	--	--			--	--	238 100%
	Respondents‘ education						Parental education					
	No formal quali-fication	8/ 9 years of school		10 years of school	12/ 13 years of school	Missing	No formal quali-fication	8/ 9 years of schooling		10 years of schooling	12/ 13 years of school	Missing
Patients	10 4.1%	42 17.2%		77 31.6%	115 47.1%	0 0%	17 7.0%	138 56.6%		54 22.1%	33 13.5%	2 0.8%
Controls	3 1.3%	55 23.1%		74 31.1%	101 42.4%	5 2.1%	16 6.7%	137 57.6%		54 22.7%	31 13.0%	0 0%
	Occupational skill level											
	First Survey						Second Survey					
Frequency %	1 (lowest)	2		3	4 (highest)	Missing	1 (lowest)	2		3	4 (highest)	Missing
Patients	8	65		32	30	109	8	66		50	83	37
	3.3%	26.6%		13.1%	12.3%	44.7%	3.3%	27.1%		20.5%	34.0%	15.2%
	5.9%*	48.2%*		23.7%*	22.2%*	--	3.9%*	31.9%*		24.2%*	40.1%*	--
Controls	6	55		53	31	93	8	73		55	62	40
	2.5%	23.1%		22.3%	13.0%	39.1%	3.4%	30.7%		23.1%	26.1%	16.8%
	4.1%*	37.9%*		36.6%*	21.4%*	--	4.0%*	36.9%*		27.8%*	31.3%*	--
	Educational **inter**generational mobility**						Occupational **intra**generational mobility**					
	-2	-1	0	+1	+2	+3	-2	-1	0	+1	+2	+3
Patients	3	14	82	77	62	4	0	6	89	22	8	0
	1.2%	5.8%	33.9%	31.8%	25.6%	1.7%	0%	4.8%	71.2%	17.6%	6.4%	0%
Controls	0	11	78	68	37	3	2	26	71	27	4	2
	0%	5.6%	39.6%	34.5%	18.8%	1.5%	1.5%	19.7%	53.8%	20.5%	3.0%	1.5%

* Percentages based only on individuals with valid information on education or occupation

** +3 denotes three steps of upward mobility from the origin; +2: two steps upward; +1: one step upward; 0: no mobility; -1: one step of downward mobility; -2: two steps of downward mobility

### Statistics

The first step of the analysis was to examine whether educational mobility from parents to respondents differed between groups by including gender and age as covariates. Analyses of educational and occupational mobility were performed with multinomial logistic regression by using intergenerational stability (no upward or downward mobility) as the reference category. An error level of p<0.5 was assumed to be acceptable for interpreting the results as significant. All statistical analyses were performed using Stata 16 MP [26]. For drawing and matching cases to controls, the additional ado-module CCMATCH was used [27].

## Results

### Patients

At the beginning of the first study, 820 patients had ever undergone surgery at the University Medical Centre Göttingen (Germany). After excluding patients with mental retardation and complex disabilities, the potential study population consisted of 698 patients. After the exclusion of patients whose addresses were unknown (13% of the gross sample) and of deceased cases (4.7%), 574 potential participants remained, which was set to 100% [22]. After 121 refusals and 92 non-responses, 360 patients had complete social data. The age range of the participants varied between 14 and 45 years. The mean age at first surgery was 7.0 years with a large standard deviation (Sd = 7.2) due to the range varying between two months and 35 years. However, 50% of patients underwent their first surgery before the age of 5, and 75% underwent their first surgery before the age of 10.

Testing for systematic attrition led to the conclusion that age, gender and cardiological variables did not differ between the 360 respondents and the 229 non-respondents [22]. For the follow-up, the list from the first study (= 364) was used as a starting point. Of these, 48 could not be tracked, and 22 patients died between the first and second wave. Finally, 294 patients were contacted successfully, the purpose of the study was explained, and they were invited to the clinic by trained personnel in charge of usual clinical routines. Forty-five explicitly declared that they did not want to participate, and two patients died after having consented to be included in the study. A total of 221 patients were examined in the clinic, 28 were interviewed only in writing, and for three patients, only medical data were collected. The data for n = 244 patients were available. An analysis of non-participants revealed that 60.3% of patients with curative surgery, 73.5% with reparative surgery, and 56.2% of patients with palliative surgery participated.

**Controls** were drawn from the Socio-Economic Panel (SOEP) according to the abovementioned criteria. The sample includes women and men who participated longitudinally (2004 and 2018) to depict changes over time. This led to a sample of n = 238 women and men; i.e., the control group was smaller than the patient group.

### Distribution of basic frequencies

Comparing the distributions of educational attainment for cases and controls (Table 1) reveals that in patients, the proportion of individuals who had not completed their education (4.1%) was higher than in the controls (1.3%), and the proportion with basic education (Hauptschule) was higher in controls (23.1% versus 17.2% in patients). A higher percentage of patients (47.1%) than controls (43.4%) had reached the highest educational level (χ^2^-test for valid cases: χ^2^(3) = 6.23; p = 0.10).

In both waves a substantial proportion of patients had missing data on their occupation (Table 1). In the first wave, this was mainly due to subjects who had not yet finished school or vocational training. Among the controls, no gender differences emerged, as 38.3% of females and 40.2% of males had missing values. Among patients, the corresponding proportions were 45.1% and 44.4%. In the patient group, disease severity was unrelated to the occurrence of missing values.

In the second wave, the number of respondents with missing values for occupation was smaller than in the first wave. Again, in the patient group, no gender differences emerged, as 14.8% of females and 15.7% of males had missing values, but now the missing data were dependent on disease severity. Compared with curatively treated patients, reparatively (OR = 9.2; 95% CI: 1.2–70.2; p = 0.03) and palliatively treated patients (OR = 13.4; 95% CI: 1.6–114.7; p = 0.02) were more likely to have missing values. Among the controls, the distributions were different, as 11.4% of males and 24.7% of females had missing values for occupation. Breaking these subgroups down by introducing sociodemographic characteristics was inconclusive due to the small number of cases, and we refrained from more detailed analyses.

### Intergenerational mobility in terms of education

**Fig 2:**
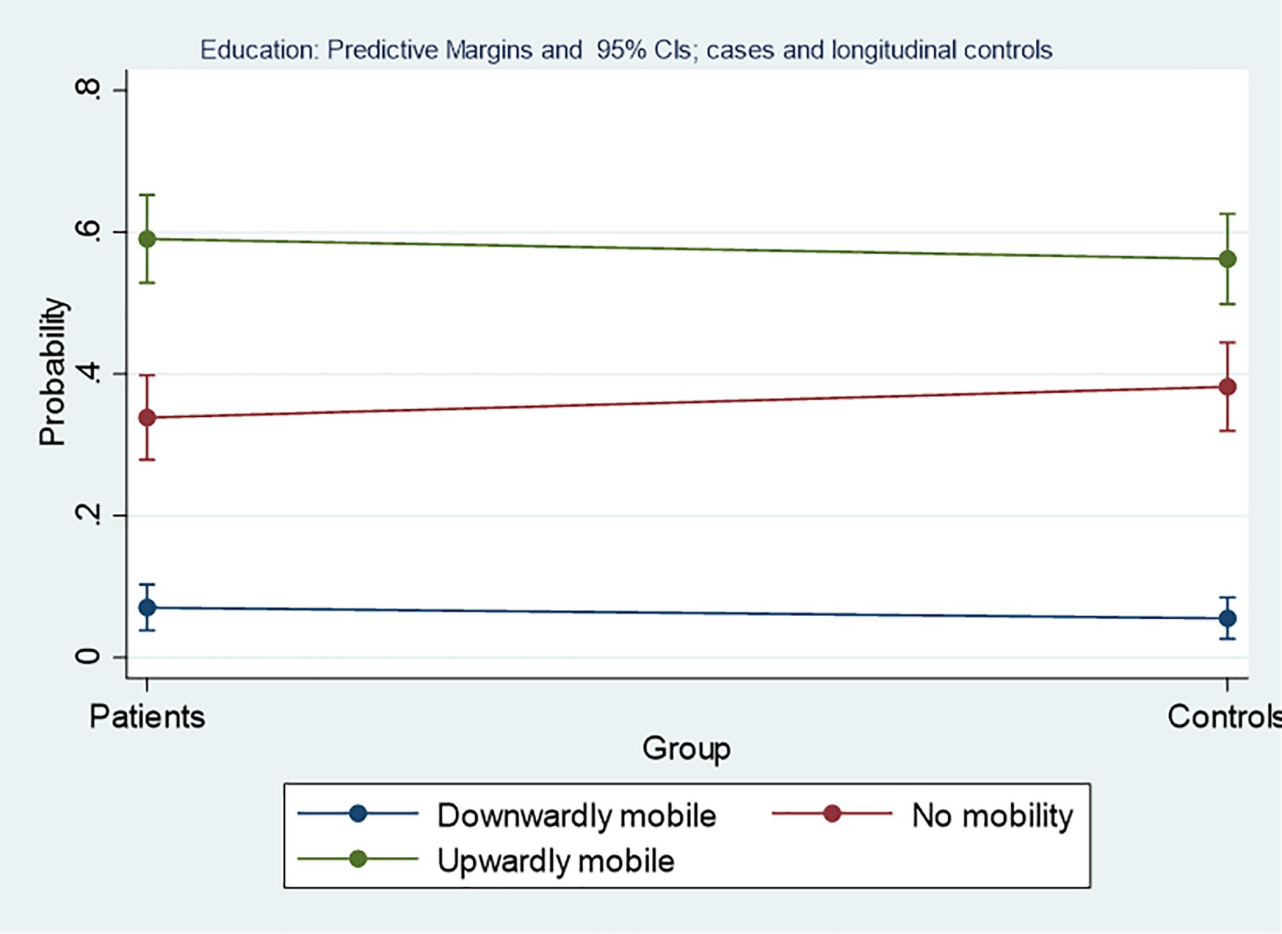
Group differences of probabilities for intergenerational social mobility in terms of educational position for patients. The y-axis is depicting the probability for falling into one of these categories with 95% confidence intervals, the x-axis depicts the two groups, i.e. cases, or controls, the horizontal line gives the slope between the group values.

Table 1 shows that in patients as well as in controls, upward and downward mobility took place. In both groups, the largest proportion was upwardly mobile, and the chances of transition decreased with increasing distance from the parental position. Multinomial regressions did not yield interpretable coefficients, as confidence intervals were large and the effects were statistically insignificant (Table 2). To clarify the findings on mobility processes, predictive margins for cases and controls were estimated (Fig 2). For SOEP controls, the probability of stability was slightly higher than for cases with congenital heart disease. Among the controls, upward mobility was somewhat lower, but in all cases, it was statistically insignificant. In the same way, no differences emerged in terms of downward mobility.

**Table 2:** Intergenerational social mobility in terms of changes of educational qualification between parental and respondent generation for cases and controls with relative risks and 95% CI.

**Variables**	RRR	StE	P	95%CI
**Downward mobility**				
Gender (female)	0.88	0.36	0.70	0.39–1.97
Group (controls)	0.70	0.28	0.36	0.32–1.52
Age (years)	1.02	0.02	0.33	0.98–1.07
Constant*	0.15	0.05	<0.01	0.07–0.30
**No mobility**	Base outcome			
**Upward mobility**				
Gender (female)	1.18	0.23	0.40	0.80–1.74
Group (controls)	0.84	0.16	0.38	0.58–1.24
Age (years)	1.00	0.01	0.89	0.98–1.03
Constant*	1.52	0.76	0.40	0.57–4.06

*Baseline relative risk for each outcome

RRR: Relative risk ratio; StE: Standard error; p: error level; 95% CI: Confidence interval

### Retirement and unemployment

Not all women and men entered the labour force and remained economically active over the whole observation period. For **retirement,** Table 3 shows that the share of individuals who left the labour force prematurely increased over time. This applies both to cases and to controls, but the proportion of retired patients was always higher. The main reason for retirement was disease severity, as in the second survey, no patient with curative surgery was retired, but 9.2% of patients with reparative, and 17% with palliative surgery, were retired. Due to the low number of cases, group differences were marginally significant in 2004 (χ^2^(2) = 3.53; p = 0.06) and were significant in 2018 (χ^2^(2) = 8.44; p<0.01).

**Table 3:** Retirement in patients and controls in frequencies, expected frequencies, and column percentages for both surveys with empirical and expected frequencies and column percentages.

	First survey		Follow-up	
Frequency Expected freq. Column-%	Patients	Controls	Patients	Controls
Not retired	236	236	223	232
	238.9	233.1	230.3	224.7
	96.7%	99.2%	91.4%	97.5%
Retired	8	2	21	6
	5.1	4,9	13.7	13.3
	3.3%	0.8%	8.6%	2.5%
Total	244	238	244	238
	100%	100%	100%	100%

For **unemployment** (Table 4), in the first survey, a lower proportion of patients (5.7%) than controls (8.4%) was unemployed, but this difference was not statistically significant (χ^2^(1) = 1.31; p = 0.25). In 2018, the situation was different, as among patients, the proportion of unemployed individuals was now higher than among controls (χ^2^(1) = 6.55; p = 0.01). In contrast to premature retirement, CHD severity was not associated with unemployment (χ^2^(2) = 1.83; p = 0.40).

**Table 4:** Distribution of unemployed patients and controls who participated in both surveys with empirical and expected frequencies and column percentages.

	First survey		Follow-up	
Frequency Expected freq. Column-%	Patients	Controls	Patients	Controls
Employed	230	218	227	233
	226.8	221.2	232.9	227.1
	94.3%	91.6	93.0%	97.9%
Unemployed	14	20	17	5
	17.2	16,8	11.1	10,9
	5.7%	8.4%	7.0%	2.1%
Total	244	238	244	238
	100%	100%	100%	100%

### Intragenerational mobility in terms of skill levels

If the first survey is taken as a starting point, a higher proportion of patients than controls started at skill levels one and two (Table 1), although taken together, the distributions for the two groups did not differ significantly (χ^2^(3) = 5.97; p = 0.11). In the next step, intragenerational mobility was examined by depicting changes in occupational positions between the two surveys. Only the data on women and men who remained economically active over the whole observation period were used (see Table 1). Again, occupational stability was used as the reference outcome in the multinomial regression.

The group effect turned out to be statistically significant, and the relative risk of being downwardly mobile was considerably higher among controls (RRR = 5.8) than among patients, while the coefficients on gender and age failed to reach statistical significance. Different from downward mobility, no significant group effect for upward mobility emerged, and all other variables also had no effect (Table 5).

**Table 5:** Occupational intragenerational social mobility for cases and controls with relative risks and 95% CI.

	Longitudinal control sample			
Variables	RRR	StE	P	95%CI
**Downward mobility**				
Gender (female)	0.82	0.33	0.63	0.38–1.80
Group (controls)	5.84	2.79	<0.01	2.29–14.88
Age (years)	1.00	0.03	0.96	0.95–1.05
Constant*	0.07	0.08	0.03	0.01–0.76
**No mobility**	Base outcome			
**Upward mobility**				
Gender (female)	0.66	0.21	0.18	0.36–1.21
Group (controls)	1.38	0.41	0.28	0.77–2.48
Age (years)	0.99	0.02	0.73	0.96–1.03
Constant*	0.53	0.47	0.48	0.09–3.01

*Baseline relative risk for each outcome

RRR: Relative risk ratio; StE: Standard error; p: error level; 95% CI: Confidence interval

**Fig 3:**
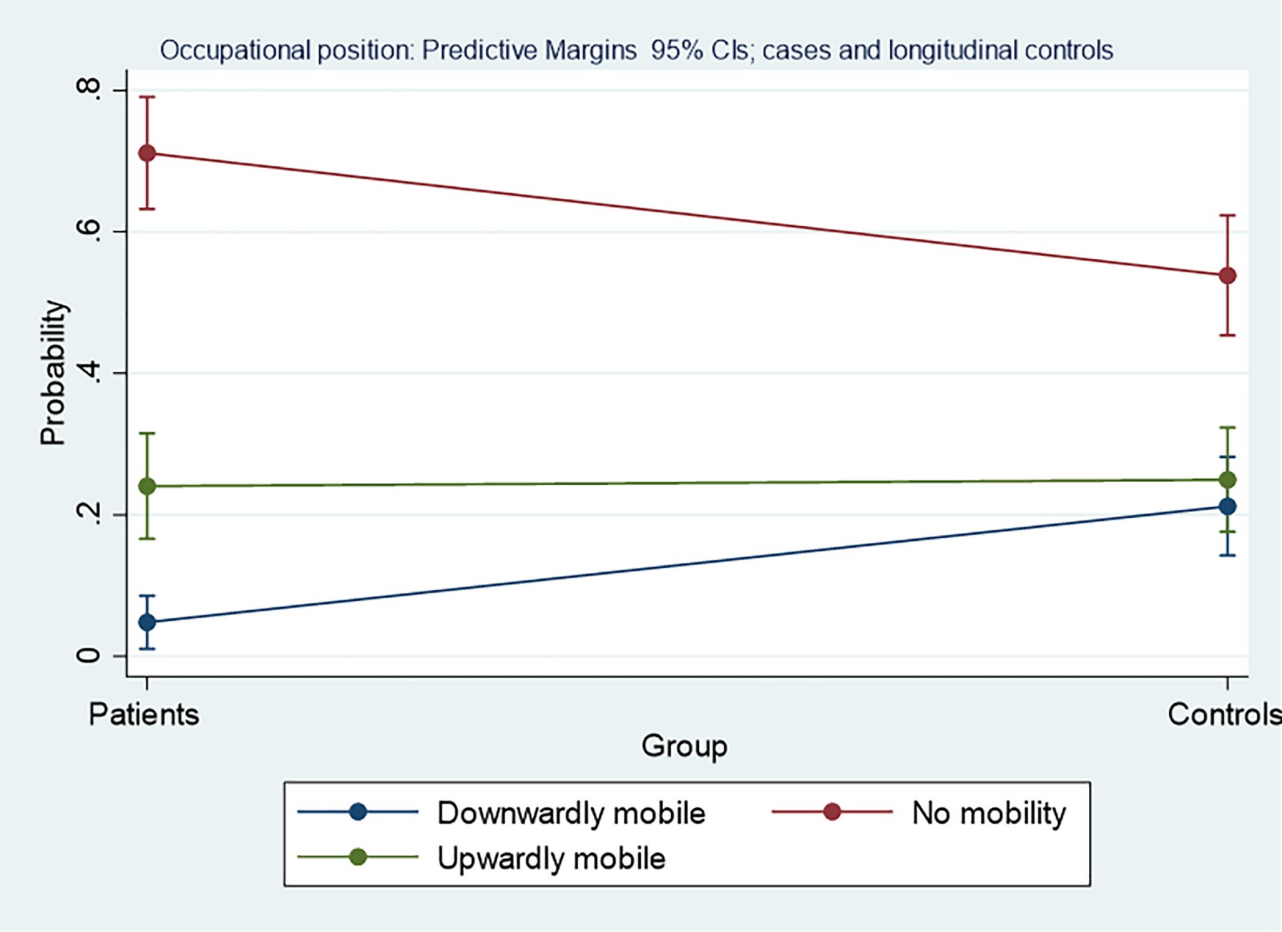
Group differences of probabilities for intragenerational social mobility in terms of occupational position for patients and for longitudinal controls. The y-axis is depicting the probability for falling into one of these categories with 95% confidence intervals, the x-axis depicts the two groups, i.e. cases, or controls, the horizontal line gives the slope between group values.

Calculating predictive margins again, the analyses show that the probability of being occupationally stable over time was higher than that of being downwardly or upwardly mobile, but stability over time was lower among controls than among patients. Among controls, this did not result in higher upward mobility but increased probability of downward mobility. In contrast, no group differences emerged for upward mobility (Fig 3).

## Discussion

This study is the first to examine life opportunities after surgery for congenital heart disease in terms of educational and occupational social mobility. It covered 15 years and included comparisons of patients and controls drawn from a national population survey. Intergenerational social mobility was depicted as upward and downward changes from the parent to the generation of patients and respondents, and educational upward mobility was found to be more common than downward changes or stability from parent to child. A core finding of these analyses was the absence of differences between patients and controls; i.e., the patterns of educational mobility were the same in both groups. An unexpected finding emerged in terms of the intragenerational mobility in occupations over 15 years. Downward mobility was more prevalent among controls than among patients, stability was the rule, but changes in the control group were mainly due to descent, and upward mobility did not differ between patients and controls. The mobility patterns found in our study samples have also been reported for Germany as a whole [28]; thus, the development of the socioeconomic positions of CHD patients is not an isolated finding. Due to the attrition rate of curatively treated patients, one may consider the upward mobility of patients to be somewhat underestimated, but the participation rate was also lower among those with palliative surgery, which may have balanced out this effect. In sum, one might be satisfied with how the situation of patients as a potentially vulnerable group has developed over time, but it should be kept in mind that these favourable findings pertain to those women and men who entered the labour market and continued to be active from the first to the second survey. In the first survey, the retirement rates of patients between 15 and 45 years of age were already higher than among controls [19], and in the second survey, the rate more than doubled when they were between 28 and 59 years old. It is most likely that this is due to disease severity, i.e., to the degree of related functional impairments.

There was also considerable unemployment (i.e., registered as seeking work) among patients, as the rate rose from 5.7% in the first survey to 7.0% in the second, but the economic conditions changed over time. In 2004, Germany had a high unemployment rate, 10.5% [29], which also affected the unemployment rate of the SOEP controls (8.4%). The economic situation improved until 2018 when the national unemployment rate was 5.2% [29], and unemployment among controls was also lower (2.1%). In that respect, the situation of individuals with congenital heart disease in Germany was different from that of the general population. Depending on the degree of impairment, patients are entitled to disability benefits, and according to German law, they are more protected from dismissal than individuals without disabilities. This does not completely preclude patients from becoming unemployed; thus, unemployment rates are difficult to interpret. In contrast to retirement, unemployment is a repeatable event, and the duration and frequency of unemployment spells that may have been experienced between the two waves or before the first wave are unknown. For that reason, our unemployment data must be interpreted as a snapshot that may nevertheless be used as an indicator for life opportunities. No studies on the effects of impaired health on social mobility are available for comparison, but for unemployment and early retirement, our findings are in line with international work [12, 14, 15, 17]. Direct numeric comparisons within and between countries are, however, difficult to draw because the reproducibility of findings is dependent on the time period when a study was conducted, on the distribution of the severity of the congenital malformations, on the quality of care, and on the welfare systems of the countries considered.

Returning to the main question of group differences in terms of social mobility, the phase of life that may signify the emergence of diverging life opportunities for patients and non-patients must be discussed. Our data point towards the end of school education, and this diversion might begin after a decision had been made regarding whether to enter the labour force or not. From a longitudinal perspective, the risk of impairments and of heart failure increase with age [2, 8] and may lead to dismissal or to the decision to leave the labour market.

In preliminary analyses, we did not find any gender differences, which is why the data for women and men were merged in the final analyses presented above. Gender was nevertheless used as a control variable to show this absence of differences without having to present separate tables for women and for men. For educational levels, the gap between women and men had long been closed in Germany. For occupational levels, it must be emphasized that we did not compare women and men with respect to their levels attained. Instead, occupational mobility from a certain point of departure was considered by disregarding gender differences in occupational levels when entering the labour market.

As not all patients in the first wave also participated in the second, the extent to which selective attrition might have affected our findings needs to be discussed. For the first wave, non-response was shown to be uncorrelated with disease severity [22]. As shown in the results section for the second survey, attrition was more frequent among patients with less severe heart defects and among those with more severe heart defects, while patients with an intermediate degree of severity were least likely to attrit. The methodological literature suggests that the explanations for non-participation for these two groups may differ. Patients who underwent curative surgery may live without complaints, and they may do so without regularly having to visit a cardiologist. In this case, interest in participating in a time-consuming study may be low, as patients may no longer feel concerned or committed to the clinic. This is supported by methodological studies reporting that interest in a study topic is the main reason for participation [30, 31]. On the other hand, response rates were reported to decline with increasing health impairments [32–34]. In particular, patients who underwent palliative surgery may have difficulties, as their cardiovascular health is at risk of deteriorating over time and participating in a study may be too exhausting. Finally, in severely impaired patients with complex congenital malformations, the final reason for non-participation was premature death. The bias resulting from these two reasons for attrition may have opposite effects. While the non-participation of patients with less severe CHD may lead to an overestimation of educational performance and the non-participation of patients with severe CHD may lead to an underestimation, the net effect is, however, difficult to assess.

Most respondents in our control group were steady participants in the SOEP. Approximately 84.5% of them took part in all 15 surveys covered by our observation period, 10.1% participated in 14 waves, 4.2% in 10, 0.8% in 12, and 0.4% in eight SOEP waves [35], and this stability has also been reported in other panel studies [36]. Nevertheless, attrition has also occurred in the SOEP, though the bulk of attrition was reported as being due to death, a change of residence or unreachability [35]. A few studies have examined more specific explanations, but only health as a reason for attrition applies to our study. In particular, poor subjective health and the presence of a chronic disease such as diabetes or a myocardial infarction were associated with non-participation [34]. As a consequence, survey participants may appear healthier than the general population. To what extent this may have caused biased results in our study has to be left unanswered, as our patients had also been affected by health impairments.

Taken together, we found that CHD patients as a group reached the same educational level as their counterparts from the general population. Once they entered the labour market, patients did equally well in terms of occupational advancement, but depending on CHD severity, a higher proportion of patients retired prematurely. The long-term outcomes of CHD surgery seem favourable overall for the majority of patients, as surgery made it possible to participate in school education as well as in their occupational life. To what extent this had effects on family life and on patients’ well-being will be the subject of further comparative investigations of our data.
